# Inclusive leadership and work-family enrichment: the roles of relational energy and power distance

**DOI:** 10.3389/fpsyg.2025.1516361

**Published:** 2025-02-05

**Authors:** Junlin Zhang, Zongrui Liu, Jianli Wang

**Affiliations:** ^1^Nanjing University of Finance and Economics Hongshan College, Nanjing, China; ^2^Jiangsu Maritime Institute, Nanjing, China; ^3^Nanjing University of Finance and Economics, Nanjing, China

**Keywords:** inclusive leadership, work-family enrichment, relational energy, power distance, positive psychology

## Abstract

Few studies have systematically examined how inclusive leadership affects employee work-family enrichment. Based on social contagion theory and the resource model of work-family enrichment, this research examined how and when inclusive leadership influences employees’ relational energy and subsequent work-family enrichment. Additionally, we examined whether power distance might influence the positive effect of inclusive leadership on relational energy. The results from a cross-sectional survey of 673 Chinese participants in Study 1 showed that inclusive leadership is positively related to work-family enrichment, and relational energy mediates the relationship between inclusive leadership and work-family enrichment. We also found that the positive effect of inclusive leadership on relational energy was greater under lower levels of power distance, as was the indirect effect of inclusive leadership on work-family enrichment via relational energy. Study 2, using three-wave data collected from 241 Chinese employees, verified the results that relational energy mediates the relationship between inclusive leadership and work-family enrichment.

## 1 Introduction

Workforce diversity—including surface-level diversity (such as age, and race, etc.) and deep-level diversity (like values, and preferences, etc.)—is increasingly viewed as a firm’s strategic priority by the managers (Thomas, 1996). However, a rising number of organizations have discovered that the potential advantages of workforce diversity cannot be obtained easily (Cook and Glass, 2014), since without leaders who actively promote and model inclusive behaviors, diversity initiatives may not be effectively implemented or sustained (Nweiser and Dajnoki, 2022). In respect to this issue, both scholars and practitioners have highlighted the significant role of inclusive leadership (hereafter, IL) playing in acquiring the potential benefits for workforce diversity (e.g., Randel et al., 2018; Korkmaz et al., 2022). IL was defined as “leaders who exhibit openness, accessibility, and availability in their interactions with followers in the workplace” (Carmeli et al., 2010, p. 250). This definition was selected for its clarity and simplicity, effectively introducing the concept of IL and underscoring the significance of approachability and interaction—key elements in establishing trust and rapport within the workplace (Javed et al., 2018). Recent studies have confirmed the positive impact of IL on various outcomes at the individual, group, and organizational levels, including voice behavior (Guo et al., 2022), creativity (Li et al., 2024), team innovation (Ma and Tang, 2023), and job performance (Randel et al., 2018). Despite these emerging insights, empirical research on the effectiveness of IL remains in its nascent stages (Randel et al., 2018). Particularly, there is limited understanding of how IL might affect work-family outcomes, such as work-family enrichment.

To advance research on the impact of inclusive leadership on employee work-family interface outcomes, we examine employees’ work-family enrichment (hereafter, WFE), defined as “the extent to which experiences in one role improve the quality of life in the other role” (Greenhaus and Powell, 2006, p. 73), as an outcome of IL. The reason for focusing on WFE is that it is an important psychological indicator for enhancing employee satisfaction, organizational performance, and societal well-being (Kalliath et al., 2019; Liu et al., 2022; Carlson et al., 2011) and its development requires the acquisition of resources from work (Greenhaus and Powell, 2006). Moreover, only two studies have examined the influence of leadership styles on WFE, including transformational leadership (Hammond et al., 2014) and servant leadership (Zhang et al., 2012). IL is a distinct leadership style from these two types: IL focuses on diversity and equality, servant leadership focuses on serving and meeting the needs of team members, and transformational leadership focuses on motivating team members to achieve higher organizational goals (Song, 2023; Jolly and Lee, 2021). Inclusive leaders may provide several important types of resources for employees to develop WFE. We can apply social contagion theory, which posits that behaviors, emotions, and attitudes can spread within a group in much the same way that a virus spreads among individuals (Barsade, 2002), to explain this relationship. Inclusive leaders often exhibit positive emotions and attitudes toward employees (Ye et al., 2018), these positive emotions can spread throughout the organization, influencing employees to feel more positive and engaged in their work. This positive emotional state can then carry over into their family lives, leading to high WFE. Therefore, the primary purpose of this paper is to examine whether IL affects employee WFE.

To gain a deeper insight into the connection between IL and WFE, it is critical to further investigate the mechanisms that underlie this relationship. Previous studies have examined the mediating variables between inclusive leadership and employee work outcomes. For example, Jiang et al. (2020) found leader-member exchange mediated the relationship between IL and voice behavior. Song (2023) demonstrated IL might increase negative feedback-seeking behavior through organizational identification. Ye et al. (2018) argued positive mood mediated the IL and employee learning from errors relationship. Although these intrapersonal mechanisms have offered profound insights, they neglect to detail the effects of IL from interpersonal perspectives. Thus, to advance the interpersonal process, this paper adopts social contagion theory (Barsade, 2002) and introduces relational energy as an interpersonal-level mechanism linking IL and WFE.

Relational energy, which is defined as “a heightened level of psychological resourcefulness generated from interpersonal interactions that enhances one’s capacity to do work” (Owens et al., 2016), reflects the energy resources one person obtains from another (Baker, 2019). Previous research indicates that relational energy often arises from positive social interactions (Wang et al., 2018). As a relational leadership style (Carmeli et al., 2010), inclusive leaders can easily establish positive social interaction relationships with employees. That is, employees may easily draw energy resources (e.g., relational energy) from the interactions with inclusive leaders. It should be noted that leader-member exchange (LMX) also represents the positive interaction relationship between leaders and employees (Graen and Uhl-Bien, 1995). Previous research has also indicated that IL can influence employee behavior through LMX (Jiang et al., 2020) and LMX is positively related to relational energy (Owens et al., 2016). To more accurately examine the mediating role of relational energy between IL and WFE, this paper controls for the influence of LMX. Based on social contagion theory (Barsade, 2002), relational energy, stemming from positive interactions with an inclusive leader, can transfer to an employee’s family life, leading to more positive and nurturing positive interactions at home, which can contribute to work-family enrichment. Thus, we are interested in whether IL affects employee WFE through relational energy, after controlling for LMX.

Previous research has indicated that the impact of inclusive leadership on employee psychology and behavior depends on the employee’s power distance (Qian and Wang, 2023), yet the conclusions are inconsistent. For example, Guo et al. (2022) argued that power distance weakened the effect of IL on leader identification; however, Ye et al. (2018) found that power distance strengthened the effect of IL on psychological safety. Therefore, it is necessary to further explore the influence of power distance on the effectiveness of IL. Power distance, which is defined as “the extent to which one accepts that power in institutions and organizations is distributed unequally” (Lian et al., 2012, p. 108), has been proven to play a critical role in the social interactive process with leaders (Song et al., 2019). Thus, we will examine how power distance moderates the relationships between IL and relational energy, and subsequent WFE. Specifically, we assume that employees with lower power distance may experience more relational energy when interacting with inclusive leaders; this is because employees with lower power distance prefer to interact with their leaders in an open and participatory manner, whereas employees with high power distance prefer to receive guidance and supervision from their leaders (Kirkman et al., 2006). Hence, we argue that inclusive leaders are more compatible with the individuals with lower power distance, thereby making relational energy and WFE more likely to be enhanced.

**Figure 1:**
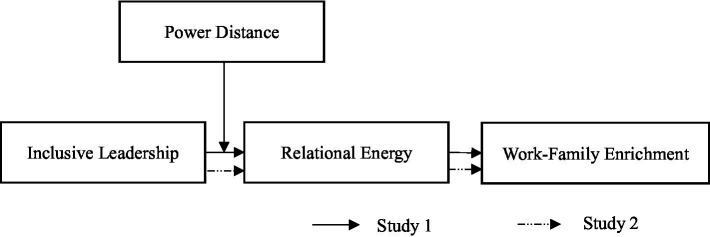
Research model.

Figure 1 illustrates our conceptual model. We conducted two studies to test the model. In study 1, we used a cross-sectional survey of 673 Chinese participants to examine the whole model. To mitigate the limitations of cross-sectional data (Podsakoff et al., 2012) and further examine the mediating effect of relational energy between inclusive leadership and work-family enrichment, Study 2 employed a three-wave dataset collected from 241 Chinese employees to test the mediating effect.

This research provides several theoretical contributions. First, by examining the influence of IL on WFE, this paper contributes to the understanding of IL’s role in enhancing work-family relationship. Previous research has indicated that IL contributes to enhancing work-related outcomes, such as positive emotions (Ye et al., 2018), psychological safety (Carmeli et al., 2010), and psychological empowerment (Javed et al., 2018). These positive psychological resources themselves spill over into the work-family interface domain (Greenhaus and Powell, 2006), thereby enhancing WFE. This extension is of considerable significance because the phenomena of WFE and IL are not only advocated but also deeply rooted in Chinese society, reflecting cultural values, economic development, social changes, and policy support that are intrinsic to the country’s progress and well-being (Tang et al., 2015; Zhang et al., 2012). This study also responds the calls for more empirical research on IL (Randel et al., 2018). Second, this study offers a novel perspective by focusing on the interpersonal mechanism to elucidate the influence of IL on WFE. Specifically, it explores the mediating role of employees’ relational energy with their leaders in the relationship between IL and WFE. Third, examining the moderating effect of power distance deepens our knowledge on the extent to which IL may impact WFE via an interpersonal relationship mechanism. By doing this, this research responds to Ye et al. (2018) call for more studies that should target the differentiation effects of IL across different cultural values.

## 2 Literature review and hypotheses development

### 2.1 IL and WFE

WFE refers to the extent to which resources gained in the work domain can enhance the quality of life in the family domain (Greenhaus and Powell, 2006). Greenhaus and Powell’s (2006) resource-based model of WFE states that the positive experiences and resources gained from work are expected to be beneficial in boosting WFE, which include skills and perspectives, referring to the experiences and abilities to successfully accomplish tasks; psychological and physical resources, referring to the positive experiences in one’s work (e.g., self-efficacy and positive emotions); social-capital resources, referring to the positive social interaction with others; flexibility, referring to the discretion to determine how and when to meet their work requirements. Moreover, the positive impact of these resources on WFE has been empirically tested (e.g., Carlson et al., 2019; Rastogi and Chaudhary, 2018). We argue that IL helps employees acquire these key resources, thereby enhancing employees’ WFE.

IL is a relational style of leadership, characterized by being good at listening to and paying attention to the needs of subordinates in an organization, and demonstrating openness, availability, and accessibility (Carmeli et al., 2010). All these characteristics enable inclusive leaders to build strong emotional connections and high-quality interpersonal relationships with employees (Ye et al., 2019), and as a result, social capital resources are enhanced. Meanwhile, IL can also help to build a supportive culture which is essential in promoting employees’ positive affect (Choi et al., 2016) and psychological security (Javed et al., 2018). Thus, IL can promote employees’ psychological and physical resources.

Prior studies also show that there may be conflicts between work and family in terms of resources and time (Lapierre and McMullan, 2015). Paying attention to employees’ interests and needs, inclusive leaders are more likely to give employees sufficient freedom and discretion to complete their tasks (Hollander, 2009; Randel et al., 2018) and deal with family affairs. Then employees can enjoy a high level of flexibility in their positions, which is crucial to develop employees’ WFE (Carlson et al., 2011). Moreover, through listening and respecting to employees’ ideas and voices, encouraging employees’ participation in decision-making, and sharing the corporate vision with employees, inclusive leaders will improve employees’ self-efficacy (Liao et al., 2010), which is one of the important sources of WFE (Rastogi and Chaudhary, 2018). Finally, inclusive leaders acknowledge and encourage different viewpoints, value diversity and equity (Nishii and Leroy, 2022), which leads to a workplace culture where employees from various backgrounds feel valued and respected. This environment encourages the sharing of unique experiences and knowledge, thereby enhancing the skills and perspectives. Thus, inclusive leaders may provide skills/perspectives resources for employees, and then WFE will be enhanced.

Building on previous discussions, we can now explore the role of social contagion theory (Barsade, 2002), which provides a framework for understanding how the positive attributes of inclusive leadership can spread and enhance employees’ WFE. This theory helps to explain how a cycle of positive behaviors, emotions, and resources fostered by inclusive leadership can transcend work boundaries and enrich family life. Inclusive leaders, by demonstrating openness, accessibility, and availability, create a ripple effect of positive behaviors and emotions that can be infected by employees. These behaviors and emotions can then be transferred from the workplace to the home environment, leading to work-to-family positive spillover. This spillover can manifest in the form of skills, perspectives, psychological and physical resources, and social capital that employees gain from their interactions with inclusive leaders. For instance, employees may adopt the same supportive and respectful behaviors they experience at work towards their family members, thus enhancing their family interactions and performance. Moreover, the positive affect promoted by inclusive leadership, such as feelings of being valued and psychologically safe, can lead to an outward focus of attention and warm, caring interactions at home, as suggested by work-family enrichment theory (Greenhaus and Powell, 2006).

*Hypothesis 1*: IL is positively related to WFE.

### 2.2 The mediating role of relational energy

Like we discussed earlier, prior research has empirically verified some inner psychological mechanisms (e.g., positive mood, psychological safety) through which IL influences employees’ job outcomes (e.g., Javed et al., 2018), but do not provide enough knowledge for us to understand how the interpersonal process may transfer the effects of IL. To enhance our understanding of the extensive effectiveness of IL, we aim to investigate an interpersonal process mechanism that may explain how IL brings benefits for WFE. Specifically, we adopt social contagion theory (Barsade, 2002) and introduce relational energy as an interpersonal mechanism that underpins the impact of IL on WFE. As suggested by social contagion theory, energy can be maintained and obtained from social interactions (Owens et al., 2016); and the spread of energy has positive effects on work and non-work outcomes (Barsade, 2002). Based on this logic, we argue that IL may promote employees’ relational energy via high-quality of social interactions; and the energy that employees get from the workplace may be easily spread to family domain, and consequently, WFE will be developed.

Relational energy originates directly or indirectly from positive leader-employee interactions (Owens et al., 2016). We argue that IL can contribute to relational energy by establishing high-quality interpersonal relationships. Specifically, inclusive leaders value employees’ contributions, show their concern for employees’ need, and are ready to offer information and resources for addressing subordinates’ troubles (Javed et al., 2018). In such a case, employees are more likely to establish and develop positive emotional connections with their leaders (Ye et al., 2018). Previous studies have also indicated that when employees can maintain positive interactive relationships with their leaders, they are more likely to experience energy (Atwater and Carmeli, 2009). When describing the dyadic relationship between leaders and subordinates, LMX is one of the most common concepts. In fact, IL is also often regarded as a typical relational leadership approach. Thus, to examine how IL affects employees’ relational energy, the impact of LMX needs to be controlled for. Distinct from LMX, which emphasizes the reciprocal relationship between leaders and subordinates (Liden and Maslyn, 1998), IL emphasizes equal and fair treatment of all employees, as well as showing care and support for every employee (Randel et al., 2018; Hollander, 2009). From this kind of relationship with inclusive leaders, employees are more likely to gain relational energy. Accordingly, we propose that IL is productively connected to employees’ relational energy.

Relational energy can produce diverse and desirable outcomes for employees (Barsade, 2002), such as job engagement (Owens et al., 2016). Greenhaus and Powell (2006) further suggest that positive psychological resources developed in the workplace would contribute to the development of WFE. Extending these logics, we argue that relational energy from the interactions with inclusive leaders will strengthen employees’ WFE by spilling over to family. Social contagion theory suggests that affective experiences and attitudes can be transferred from one person to another (Barsade, 2002) and even across organizational boundaries (Bal and Boehm, 2017). Relational energy can enhance cognitive flexibility (Owens et al., 2016), which is the ability to switch between different cognitive tasks or perspectives. This flexibility can be contagious, helping employees to navigate the complex demands of work and family life with greater ease (Greenhaus and Powell, 2006). Moreover, the time and resources that employees devote to family and work matters are finite (Russo et al., 2018). When more time and resources are invested in work, less is available for family affairs. Previous research has indicated that employees with high relational energy can achieve higher work performance with less time and resource investment (Owens et al., 2016; Wang et al., 2018). This means that employees with high relational energy are more likely to have sufficient time and resources to devote to family matters, thereby performing more effectively (Ten Brummelhuis and Bakker, 2012; Wayne et al., 2007). As such, we propose:

*Hypothesis 2:* Relational energy mediates the positive relationship between IL and WFE.

### 2.3 The moderating role of power distance

Social contagion theory suggests that individual values have a significant impact on the transmission of emotions and energy between individuals (Bakker and Schaufeli, 2000). As an important kind of culture value, power distance plays a significant role in how employees react to a certain kind of leadership (Song et al., 2019; Farh et al., 2007). Therefore, we contend that power distance will moderate the effect of IL on relational energy. Specifically, we suggest that employees who demonstrate a lower level of power distance may react to IL in a more positive manner and thus experience a higher level of relational energy.

As proposed, power distance is expected to indicate the degree to which an employee may accept the unequal distribution of power within the organization (Lian et al., 2012). Employees with a higher level of power distance would expect their leaders to command direct instructions to them about what needs to be done (Kirkman et al., 2009). When inclusive leaders provide employees with more opportunities to communicate coequally (as counterparts) and invite employees to convey their opinions and suggestions, employees with a higher level of power distance will feel uncomfortable (Madlock, 2012). Consequently, when leaders treat employees in a more inclusive manner, they will experience a lower level of relational energy with leader because IL may not be compatible with their high level of power distance. Comparatively, employees with a lower level power distance may resonate with the pleasure of IL, thereby experiencing a higher relational energy in interactions with inclusive leaders, because these employees are more willing to communicate with leaders on an equal footing (Kirkman et al., 2009).

In addition, employees with a higher level of power distance may face the dilemma of developing and benefiting from a personal and social relationship with their leader because they would be apt to take the relationship as subordinates and superiors rather than equal ones and thus maintain a high level of social distance with their leaders (Farh et al., 2007). The equal and individualized relationship with a leader is not considered as being precious by employees with a higher level of power distance who are less likely to be positively swayed by IL since it violates the rules of affiliation (Lian et al., 2012). Therefore, IL may be not consistent with employees’ higher power distance, which may bring these employees disturbed experiences and even impose a negative effect on their leaders (Gross and John, 2003). Conversely, employees with lower level of power distance favor to create social bond with their leaders. When leaders treat them inclusively, these employees are more likely to establish high-quality relationship with their inclusive leaders, and then experience higher relational energy. All of the above evidence that IL will have a potent influence on relational energy when one’s power distance level is lower. Hereby, we propose:

*Hypothesis 3:* Power distance indeed moderates the relationship between IL and relational energy in a way that the relationship is much stronger for employees with a lower level of power distance.

Based on these discussions and analyses, we propose a moderated mediation model—power distance moderates the mediating effect of relational energy in the relationship between IL and WFE. As we have contended that equal and open communication is favored by employees with a lower level of power distance, IL might enjoy a more harmonious relation with employee at a low level of power distance. Accordingly, we predict that the mediating influence of relational energy on the connection between IL and WFE will be more pronounced for employees with lower level of power distance.

*Hypothesis 4*: The relationship between IL and WFE mediated by relational energy is expected to be stronger for employees with lower level of power distance.

### 2.4 The present studies

This research employs two studies to test the model. Study 1 utilizes cross-sectional data to examine the entire model. Given the limitations of cross-sectional data in testing the relationships between variables, Study 2 uses a three-wave longitudinal research design to test the mediating effect of relational energy in the relationship between IL and WFE. This approach has been applied in previous studies (Jiang et al., 2020).

## 3 Study 1

### 3.1 Sample and procedures

The participants in this research were full-time employees who were recruited via alumni networks from three large universities in China. This method to collect data was widely adopted in prior research (Qin et al., 2018), which suggested that it is effective and feasible. Before answering the questionnaires, the participants were invited to read the explanatory statements (i.e., participation was voluntary, all the information was confidential, and only for research purpose). To ensure the respondents met our requirements, like in prior research (Jiang et al., 2020), we further designed an extra item at the beginning of the survey like “Are you a full-time worker in a firm?,” If “Yes,” the survey was continued; If “No,” the survey was completed.

The researchers sent out a total of 1,000 questionnaires and 731 participants completed them. After deleting incomplete questionnaires, we finally obtained 673 valid questionnaires. The final response rate was 67.3%. Among these samples, 53.9% were female. Regarding age, 13.5% were under the age of 25; 27.6% were between 25 and 30 years old; 20.2% were between 31 and 35 years old; 20.7% were between 36 and 40 years old; 18% were over 40 years old. In terms of organizational tenure, 6.7% were less than 1 years; 14.6% were between 3 and 5 years; 24.4% were between 6 and 8 years; 54.3% were more than 8 years. Additionally, 499 employees (74.1%) had a bachelor degree, and the rest (25.9%) had a postgraduate degree.

### 3.2 Measurement

#### 3.2.1 IL

We measured IL using the 9-item scale developed by Carmeli et al. (2010). A sample expression is “my supervisor is ready to listen to my requests.” The Cronbach’s alpha value of this scale is 0.87.

*Relational energy.* Employees’ relational energy was measured by a 5-item scale developed by Owens et al. (2015). A sample item is “I feel increased vitality when I interact with my supervisor.” The Cronbach’s alpha value of this scale is 0.89.

#### 3.2.2 WFE

We adopted Carlson et al. (2006) 9-item scale to measure WFE. An example of item is “My engagement in my job helps me understand diverse perspectives, which in turn makes me a better family member.” The Cronbach’s alpha value of this scale is 0.88.

#### 3.2.3 Power distance

Power distance was measured using a 6-item scale created by Dorfman and Howell (1988). A sample item is “Managers ought to make the majority decisions without consulting subordinates.” The Cronbach’s alpha value of this scale is 0.88.

#### 3.2.4 Control variables

Prior research has demonstrated that WFE may be influenced by some demographic variables, such as gender, education, and organizational tenure (Greenhaus and Powell, 2006). In this study we controlled for these variables. LMX was also controlled for because it has been argued that LMX positively related to favorable work-family experiences (Lapierre et al., 2018). LMX was measured by a 7-item scale developed by Graen and Uhl-Bien (1995). A sample item is “my supervisor is clearly aware of my job challenges and needs.” The Cronbach’s alpha value of this scale is 0.87.

### 3.3 Results

Considering the data is cross-sectional and from a single source, we conduct Harman’s one-factor test to examine common method bias. The results show that the first factor’s explanatory power is 31.7%, which is within the acceptable range (Podsakoff et al., 2003). Before examining the hypotheses, confirmatory factor analysis (CFA) was also conducted to ensure that our key variables (IL, WFE, relational energy, power distance, and LMX) had favorable discriminant validity. The CFA results (see Table 1) indicated that the hypothesized five-factor model fit noticeably better than any other alternative models (χ^2^ (584) = 1442.80, CFI = 0.93, TLI = 0.92, RMSEA = 0.05), supporting this discriminant validity of our variables in this study.

**Table 1:** Results of confirmatory factor analysis of study 1.

Model	x^2^	df	TLI	CFI	RMSEA
Five-factor model: IL; RE; PD; WFE; LMX	1442.80	584	0.92	0.93	0.05
Four-factor model: IL + RE; PD; WFE; LMX	2928.28	588	0.79	0.80	0.08
Three-factor model: IL + RE; PD + WFE; LMX	5016.40	591	0.61	0.63	0.10
Two-factor model: IL + RE; PD + WFE + LMX	7062.97	593	0.43	0.46	0.13
One-factor model: IL + RE + PD + WFE + LMX	8604.10	594	0.29	0.33	0.14

*N = 673. IL, Inclusive leadership; RE, Relational energy; PD, Power distance; WFE, work-family enrichment; LMX, Leader-member exchange.*

We also calculated the constructs average variance extracted (AVE) scores. The AVE scores range from 0.53 to 0.71, all of which are all higher than 0.50 (Hair et al., 1992). Moreover, all the square root scores of AVE exceeded the correlation coefficients between the variables, suggesting that these constructs demonstrate a notably high level of discriminant validity. Hence, the discriminant validity of all the proposed constructs in this research is verified.

The means, standard deviations, and correlations of the variables are illustrated in Table 2. IL is positively associated to relational energy (*r* = 0.38, *p* < 0.01) and WFE (*r* = 0.25, *p* < 0.01). Relational energy is also related to WFE (*r* = 0.25, *p* < 0.01).

**Table 2:** Descriptive statistics and correlations of study 1.

Variable	M	SD	1	2	3	4	5	6	7	8
1. Age	3.02	1.32								
2. Gender	0.46	0.50	−0.01							
3. Education	1.74	0.44	−0.20**	−0.24**						
4. Tenure	3.56	1.23	0.67**	−0.03	−0.17**					
5. LMX	3.55	0.64	0.03	0.07	0.03	0.04				
6. Inclusive leadership	3.79	0.59	0.04	0.01	0.01	0.07	0.28**			
7. Relational energy	3.62	0.78	0.04	0.02	−0.02	0.10*	0.23**	0.38**		
8. Power distance	3.36	0.91	0.07	−0.01	0.02	0.11**	0.01	0.17**	0.23**	
9. WFE	3.60	0.68	0.06	0.07	−0.01	0.07	0.27**	0.25**	0.25**	0.08*

*N* = 673; *p < 0.05; **p < 0.01.

A regression analysis was conducted to test Hypothesis 1. As shown in Table 3, after controlling for the demographic variables (e.g., age, gender, education, and tenure) and LMX, IL significantly and positively predicted WFE (*β* = 0.19, *p* < 0.01, M5). Hypothesis 1 was supported.

**Table 3:** Regression results of study 1.

	Relational energy			WFE	
	Model 1	Model 2	Model 3	Model 4	Model 5
Age	−0.05	−0.04	−0.06	0.02	0.03
Gender	0.01	0.01	0.01	0.06	0.06
Education	−0.02	−0.02	−0.02	0.02	0.02
Tenure	0.12*	0.10*	0.10*	0.05	0.04
LMX	0.23**	0.14**	0.14**	0.25**	0.20**
Inclusive leadership		0.34**	0.25**		0.19**
Relational energy					
Power distance			0.18**		
Inclusive leadership * Power distance			−0.16**		
R^2^	0.06	0.17	0.22	0.07	0.11
F	9.14	22.60	23.16	10.37	13.19

*N* = 673. *p < 0.05; **p < 0.01.

Hypothesis 2 proposed that relational energy mediated the relationship between IL and WFE. A Monte Carlo mediation test was utilized to estimate the confidence interval for the mediating effect of relational energy. The results indicated that the indirect effect from IL to WFE, mediated by relational energy, was statistically significant (*B* = 0.06, boot SE = 0.02, 95% CI = [0.03, 0.10]). Hypothesis 2 was supported.

**Figure 2:**
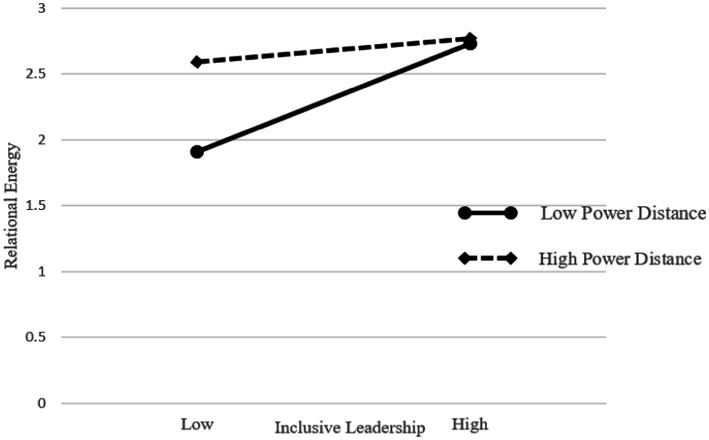
The moderating effect of power distance.

Hypothesis 3 argued that power distance moderated the positive relationship between IL and relational energy. The results from Table 3 demonstrated that the interaction of IL and power distance was negatively correlated with relational energy (Model 3, β = −0.16, p < 0.01). Using Aiken and West’s (1991) procedure, we further illustrated the interaction effect. As Figure 2 showed, for employees with a lower power distance (1 standard deviation below the mean), IL had a greater influence on relational energy. Hypothesis 3 was supported.

Hypothesis 4 proposed that the indirect influence of IL on WFE via relational energy might be moderated by power distance. The results using PROCESS (Hayes, 2013) suggested that the indirect effect of relational energy was statistically significant when employees’ power distance was low (estimate = 0.07, boot SE = 0.02, 95% CI = [0.03, 0.12]) but insignificant when it was high (estimate = 0.02, boot SE = 0.01, 95% CI = [−0.01, 0.04]). The index of moderated mediation was −0.03 (95% CI = [−0.05, −0.01]). In addition, these two conditional indirect effects were significantly different from each other (difference [high minus low] = −0.05, 95% CI [−0.10, −0.02]). Hypothesis 4 was supported.

## 4 Study 2

Study 1 utilizes cross-sectional data to test the mediating effect of relational energy between IL and WFE, and to address common method bias, Study 2 employs three-wave data to test this mediating effect.

### 4.1 Sample and procedures

Data was obtained through conducting a field questionnaire survey in a large manufacturing enterprise in eastern China, with the support of its CEO. Before the survey, we contacted the human resource manager of the enterprise, facilitated by the CEO, and requested that he/she provide the employee roster. This allowed the researchers to match the questionnaires administered to the same participants at different stages. Each participant was informed that participation in the survey was voluntary and solely for academic research purposes, and that all information they provided would be kept strictly confidential.

In order to reduce the impact of common method bias, the data were collected in three waves. At Time 1, employees were required to provide their demographic information (including age, gender, education, and organizational tenure) and rate IL and LMX. A total of 350 questionnaires were issued and 291 were returned. At Time 2 (3 months after Time 1), we distributed relational energy questionnaires to those 291 employees and received 277 questionnaires. At Time 3 (3 months after Time 2), WFE questionnaires were administered to the employees who participated at Time 2, and 255 questionnaires were recovered. After deleting incomplete questionnaires, we finally obtained 241 valid questionnaires.

Of the 241 employees, 68.5% were female. 11.2% were under the age of 25; 23.2% were between 25 and 30 years old; 27.8% were between 31 and 35 years old; 31.5% were between 36 and 40 years old; and 6.2% were over 40 years old. In terms of organizational tenure, 12.0% were less than 1 year; 33.2% were between 3 and 5 years; 12.9% were between 6 and 8 years; and 6.6% were more than 8 years. In addition, 91 employees (37.8%) held a bachelor’s degree, while 18.3% held a postgraduate degree.

### 4.2 Measurement

We used the same scales as in Study 1 to assess IL (at Time 1), relational energy (at Time 2), WFE (at Time 3), and LMX (at Time 1). The Cronbach’s alphas for these variables were 0.89, 0.90, 0.87, and 0.88, respectively.

### 4.3 Results

The results of Harman’s one-factor test indicated that the first factor’s explanatory power was 27.9%, which was within the acceptable range (Podsakoff et al., 2003). The results of CFA (Table 4) suggested that the hypothesized four-factor model fitted the data well (χ^2^ (399) = 712.87, CFI = 0.90, TLI = 0.91, RMSEA = 0.06) and better than the other three alternative models, supporting the discriminant validity of our measures.

**Table 4:** Results of confirmatory factor analysis of study 2.

Model	x^2^	df	TLI	CFI	RMSEA
Four-factor model: IL; RE; WFE; LMX	712.87	399	0.90	0.91	0.06
Three-factor model: IL + LMX; WFE; RE	1444.63	402	0.68	0.71	0.10
Two-factor model: IL + LMX; WFE + RE	2003.81	404	0.52	0.55	0.13
One-factor model: IL + RE + WFE + LMX	2481.35	405	0.37	0.42	0.15

*N = 241. IL, Inclusive leadership; RE, Relational energy; WFE, Work-family enrichment; LMX, Leader-member exchange.*

The means, standard deviations, and correlations of the research constructs are presented in Table 5. As anticipated, both IL (*r* = 0.29, *p* < 0.01) and relational energy (*r* = 0.31, *p* < 0.01) are positively related to WFE.

**Table 5:** Descriptive statistics and correlations of study 2.

Variable	M	SD	1	2	3	4	5	6	7
1. Age	2.98	1.12							
2. Gender	0.31	0.46	0.23**						
3. Education	1.74	0.75	−0.11	−0.08					
4. Tenure	2.69	1.06	0.28**	0.13*	−0.01				
5. LMX	3.49	0.64	−0.06	0.01	−0.03	0.09			
6. Inclusive leadership	3.80	0.59	−0.11	−0.06	0.01	−0.05	0.26**		
7. Relational energy	3.57	0.79	−0.09	−0.14*	0.11	−0.04	0.24**	0.37**	
8. WFE	3.58	0.70	−0.16*	−0.06	0.09	−0.15*	0.35**	0.29**	0.31**

*N* = 241; *p < 0.05; **p < 0.01.

Hayes (2013) PROCESS macro for SPSS with 5,000 bootstrap samples. The findings (see Table 6) showed that IL was positively correlated with WFE (*β* = 0.17, SE = 0.07, *p* < 0.01). Hence, Hypothesis 1 was supported. The result also showed that the indirect effect was statistically significant from IL to WFE via relational energy (*B* = 0.05, boot SE = 0.03, 95% CI = [0.01, 0.13]). Thus, Hypothesis 2 was supported.

**Table 6:** Regression results of study 2.

	Relational Energy		WFE	
	B	SE	B	SE
Age	−0.01	0.04	−0.04	0.04
Gender	−0.09	0.09	0.01	0.08
Education	0.08	0.06	0.07	0.05
Tenure	−0.02	0.05	−0.09	0.04
LMX	0.20**	0.07	0.31**	0.07
Inclusive leadership	0.43**	0.08	0.17*	0.07
Relational energy			0.15*	0.06
R2	0.06	0.17	0.07	0.11
F	9.14	22.60	10.37	13.19

*N* = 241. *p < 0.05; **p < 0.01.

## 5 Discussion

IL has attracted increased attention in both academia and practice. However, limited research has specifically investigated the impact of IL on work–family outcomes. We aim to fill this gap by integrating IL and WFE literature to examine how and when IL influences WFE. Our results demonstrate that IL is positively related to WFE. Relational energy partially mediates the relationship between IL and WFE. Furthermore, power distance negatively moderates the effect of IL on relational energy. We also find that the mediating mechanism of relational energy between IL and WFE is more prominent when employees have lower power distance rather than higher.

### 5.1 Research contributions

Several key contributions are expected to be made in this article. First, we expand the effects of IL from the work domain to the work-family interface, contributing to the IL literature. Extant studies have largely examined how IL affects employees’ job-related outcomes such as innovative work behavior (Carmeli et al., 2010; Javed et al., 2017), work performance (Hirak et al., 2012; Mitchell et al., 2015), and voluntary turnover (Nishii and Mayer, 2009; Randel et al., 2018). By identifying and examining WFE as an important representative of work–family outcomes influenced by IL, this study extends this line of research. In addition, this study responds to the call from Javed et al. (2018) for more empirical research on the effectiveness of IL.

Second, our research widens the scope of psychological consequences of IL by introducing relational energy as an important mediation mechanism. Most existing research examining the underlying mechanisms of the correlations between IL and individual outcomes has concentrated more on employees’ intrapersonal states (Choi et al., 2017; Javed et al., 2017) and less on interpersonal processes. Our study suggests that relational energy, which is a key dyadic interaction mechanism, may mediate the relationship between IL and WFE. This process may provide new insights into revealing the impact of IL and, to some extent, respond to the urge from Javed et al. (2018) to adopt new perspectives and identify new potential mediating mechanisms to unveil the influences of IL on individual outcomes.

Third, by incorporating power distance as a moderator into the relationship between IL and relational energy, this research shifts the boundary of IL from personal socio-demographic characteristics (Ye et al., 2018) to deeply-rooted cultural values. The results suggest that how IL influences relational energy may depend on employees’ power distance. These findings not only provide additional support for the argument that the influence of social interaction on individuals’ affects and attitudes is not well adapted to individuals with a higher power distance (Farh et al., 2007; Lee et al., 2000), but also acknowledge the importance of paying much more attention to the moderating impacts of cultural value orientations on reactions to inclusive leaders (Ye et al., 2018).

Lastly, this study also broadens the existing literature on WFE by exploring IL as a precursor to WFE. Although prior research (Major and Morganson, 2011; Russo et al., 2018) has provided numerous implications that leadership-related factors are essential in predicting WFE, only a few studies have examined the effectiveness of leadership on WFE, in which transformational leadership (Hammond et al., 2014) and servant leadership (Zhang et al., 2012) have been examined. As discussed above, IL is conceptually different from those leadership styles. Our discovery extends this area of research by explicitly demonstrating that IL significantly fosters employees’ WFE.

### 5.2 Practical implications

Our study offers several crucial implications for organizations. First, the research findings indicate that IL contributes to employees’ WFE. To enhance employees’ WFE, IL among formal or informal leaders at various levels of the organizational hierarchy should be encouraged. For example, offering training and development programs on IL to leaders at all levels, as well as an effective incentive and reward or promotion system, will make leaders more inclined to practice IL behaviors. Second, our findings indicate that relational energy mediates the positive relationship between IL and WFE. To foster and enhance employees’ WFE, organizations or managers should recognize the importance of relational energy and lay a solid foundation to help employees enhance their relational energy with leaders. For example, encouraging leaders to demonstrate openness and approachability in their interactions with subordinates, value the needs of their subordinates, and establish a good dual relationship with them (Nishii and Leroy, 2022). Third, the research findings have demonstrated the moderating role of power distance in the relationship between IL and relational energy, and subsequently WFE. This suggests that leaders should consider employees’ power distance when displaying IL. For instance, for those individuals who hold a higher power distance, leaders should adopt other strategies (e.g., direct guidance and help, Kirkman et al., 2009) to promote higher levels of WFE.

### 5.3 Limitations and future directions

A few limitations of this study should be mentioned. To start with, all data were gathered through self-reports, raising concerns about common method variance. Future studies should obtain data from a wide variety of sources. For example, employees’ WFE could be assessed by their family members, and IL could be assessed through self-evaluation by leaders. Given that the data in Study 1 were collected at a single time point, we cannot definitively establish causality in the relationships related to IL, relational energy, and WFE. Although Study 2 used data from three time points, reducing the likelihood of common method bias, this still cannot establish a strictly causal relationship. Future research could utilize experience sampling methods and experimental or quasi-experimental designs to establish causality and assess how the constructs change over time.

Moreover, our results demonstrate that relational energy plays a partial mediating role in the relationship between IL and WFE after controlling for LMX, suggesting that there may be additional mediation mechanisms to be discovered and examined. Existing research indicates that IL contributes to enhancing employees’ identification with their leaders or the organization (Song, 2023). Future studies could explore the mediating effects of different forms of social identification—such as organizational or leader identification—on the relationship between IL and WFE.

Lastly, this research only examined the moderating role of power distance in the relationship between IL and relational energy; however, other personal and situational factors might also influence the effectiveness of IL. Previous studies (e.g., Hollander, 2009) have indicated that the impact of a leadership style on employees may be influenced by factors such as job characteristics, organizational climate, and/or culture. Future research can further investigate the impact of these potential factors, such as organizational climate, on the effectiveness of IL.

## Data Availability

The original contributions presented in the study are included in the article/supplementary material, further inquiries can be directed to the corresponding author.
